# hsa_circ_0139402 Promotes Bladder Cancer Progression by Regulating hsa-miR-326/PAX8 Signaling

**DOI:** 10.1155/2022/9899548

**Published:** 2022-02-01

**Authors:** Bo Wei, Zunxian Wang, Qixin Lian, Baojin Chi, Shuxia Ma

**Affiliations:** ^1^Basic Medical College, Jiamusi University, Jiamusi Heilongjiang 154007, China; ^2^Department of Urology, The First Affiliated Hospital of Jiamusi University, Jiamusi Heilongjiang 154003, China; ^3^Department of Radiotherapy and Chemotherapy, The First Affiliated Hospital of Jiamusi University, Jiamusi Heilongjiang 154003, China; ^4^Department of Oncology, The First Affiliated Hospital of Jiamusi University, Jiamusi Heilongjiang 154003, China

## Abstract

**Background:**

Bladder cancer (BC) is a malignant and common malignant tumors. However, the prognosis of most patients with bladder cancer is still poor, and it is particularly important to identify early tumor diagnostic and treatment targets.

**Materials and Methods:**

High-throughput sequencing was used to evaluate the expression level of circRNA in bladder cancer tissue. MTT assay, wound healing assay, and transwell assay were used to detect the cancer cells' proliferation, migration, and invasion affected by hsa_circ_0139402. The possible miRNA targets of hsa_circ_0139402 and downstream genes were detected by bioinformatics methods and dual-luciferase reporting experiment. FISH was used to observe their interaction.

**Results:**

High-throughput sequencing result showed that the expression of hsa_circ_0139402 was highest in BC tissues and increased in metastatic tissues compared to that of nonmetastatic tissues. MTT assay, wound healing assay, and transwell assay revealed that sh-hsa_circ_0139402 could suppress BC cells' proliferation, invasion, and migration. Bioinformatics analysis, dual-luciferase reporter, and RIP assay showed that hsa_circ_0139402 can bind to hsa-miR-326, and PAX8 is a direct target of hsa-miR-326 in BC cell. Further, cytological studies found that hsa_circ_0139402 enhances BC cells' proliferation, migration, and invasion by targeting PAX8 via hsa-miR-326.

**Conclusion:**

hsa_circ_0139402 plays a oncogene in BC and that can effectively promote cell proliferation, migration, invasion, and EMT by targeting Paired Box Protein Pax-8 (PAX8) via hsa-miR-326 and provides a potential therapeutic target for BC patients.

## 1. Introduction

Bladder cancer (BC) is a malignant tumor that occurs on the mucous membrane of the bladder and is one of the most common malignant tumors. Globally, the incidence of bladder cancer is at 9th and cancer-related deaths is at 13th in all cancer [1]. Although surgery combined with chemotherapy and radiotherapy improves the prognosis to some extent, the prognosis of most patients with muscle invasive bladder cancer is still poor. In order to improve the clinical efficacy and prognosis of patients with bladder cancer and to clarify the pathogenesis of bladder cancer, it is particularly important to identify early tumor diagnostic markers and biological treatment targets.

Circular RNA (circRNA) is different from general linear RNA. It has a closed circular structure, is highly insensitive to nucleases, and has strong stability [2–4]. Studies have shown that the functions of circRNA have regulated transcription and RNA splicing. As a competitive endogenous RNA, it combines with miRNA to play the role of miRNA molecular sponge and regulate the expression of target genes in cells [5–7]. More and more evidences show that circRNA is highly correlated with a variety of tumor characteristics, including tumor development, metastasis, angiogenesis, proliferation, and invasion [8]. Some studies have shown that some circRNAs are dysregulated in bladder cancer and can promote the progression of bladder cancer, such as circRIP2, circACVR2A, hsa_circ_0001361, and circMETTL3, and could affect bladder cancer cells' proliferation and metastasis [9–12]. At present, noninvasive urine detection, methylation detection products, and new molecular biomarker (circRNA and lncRNA) maybe the new therapeutic targets and the future development trends in bladder cancer [13]. It is suggested that circRNA can be used as a good biomarker and therapeutic target for early diagnosis and prognosis of bladder tumors.

In this experiment, we collected clinical samples from bladder cancer patients to screen out differentially expressed circRNA molecules. It was found that hsa_circ_0139402 was highly expressed in bladder cancer. This experiment verifies that hsa_circ_0139402 sponges adsorb hsa-miR-326 to regulate the translation of related proteins through in vivo and in vitro experiments, which affects the occurrence and development of BC and provides experimental evidence for the discovery of molecular markers for the diagnosis, treatment, and prognosis of BC.

## 2. Materials and Methods

### 2.1. Clinical Specimens

BC clinical specimens and adjacent normal tumors were collected from the First Affiliated Hospital of Jiamusi University. This study was approved by the Ethics Committee of the First Affiliated Hospital of Jiamusi University, so the clinical sample information was informed to the patient before the operation, and the patient's consent was obtained. All of the clinical specimens were immediately flash-frozen in liquid nitrogen during surgery until RNA extraction.

### 2.2. Bioinformatics Analysis

Four online databases (circbase, circinteractome, starbase, and circbank) were used to find the potential miRNA of hsa_circ_0139402.

### 2.3. Cell Culture and Cell Transfection

UMUC3, UMUC3, 5637, J82, and SV-HUC-1 were purchased in the Cell Collection Committee of the Chinese Academy of Sciences (Shanghai, China). Cells were incubated by RPMI-1640 medium (Hyclone, USA) with 10% FBS (ExCell, China) at 37°C, 5%CO_2_. The cell lines were identified by the Short Tandem Repeat (STR) method (Biowing, China). shRNA and overexpression of hsa_circ_0139402 were obtained from GenePharma company (Shanghai, China). hsa-miR-326 mimics were purchased from RiboBio (Guangzhou, China), and cell transfection was performed using Lipofectamine 2000 (Invitrogen, USA) according to the manufacturer's protocol.

### 2.4. RNA Isolation, RNase R Treatment, and Real-Time PCR

Bladder cancer specimens were removed from liquid nitrogen tank, cut it up, and added TRIzol reagent (Invitrogen, CA, USA). Total RNAs were isolated from the paired tissue samples of bladder cancer. Then, RNA was sent to a gene testing company (biomarker, http://www.biomarker.com.cn/) for high-throughput sequencing. 2 U/*μ*g RNase R (Epicentre Technologies) was used to treat 5 *μ*g total RNA 30 min at 37°C. cDNA was synthesized by random primers and stem-loop primers with TaKaRa system (Takara, Dalian, China). RT-PCR primers were purchased from RiboBio. hsa_circ_0139402—forward: CCCCTGTACGAAGTGGACA and hsa_circ_0139402—reverse: TATCCAGTCTCCTG-TCCTCGC; hsa-miR-326—forward: CATCTGTCTGTTGGGCTGGA and hsa-miR-326—reverse: AGGAAGGGCCCAGAGGCG; PAX8—forward: GGTGGGGTCATGTGT-GTGG; BCL—reverse: CGGTTCAGGTACTCAGTCATCC; and *β*-actin—forward: CTT-AGTTGCGTTACACCCTTTCTTG and *β*-actin—reverse: CTGTCACCTTCACCGTTC CAGTTT. Real-time PCR was detected by the CFX96 Tm Real-Time System (Bio-Rad, USA). The calculation method of relative expression was the 2^−*ΔΔ*Ct^ method.

### 2.5. MTT Assay

2 × 10^3^ cells/well 5637 and UMUC3 cells were injected into 96-well plates. After 24 h, 10 *μ*L of MTT solution (Yeasen, China) was combined and incubated without light for 2 h-4 h at 37°C. The absorbance was measured at a wavelength of 490 nm using the BioTek (Winooski, USA) microplate spectrophotometer.

### 2.6. Wound Healing Assay

2 × 10^3^ cells/well 5637 and UMUC3 cells were injected into 6-well plates. 24 h later, the cell monolayer was scratched by a 200 *μ*L pipette, and the fresh medium without FBS was used to wash the plates three times. After 48 h, the wound width was calculated by the Image J software.

### 2.7. Transwell Assay

5 × 10^3^ cells/well 5637 and UMUC3 cells were injected into the upper chambers with 100 *μ*L base medium, and the lower chambers were added into medium containing 10% FBS. After 48 h, the chamber was taken out, then discarded the culture medium, washed it twice with PBS, fixed it with formaldehyde for 30 min, dyed it with 0.1% crystal violet for 30 min, and counted the cells under the microscope.

### 2.8. Dual-Luciferase Reporter Assay

The sequence of hsa_circ_0139402 was cloned into vector. Mutations were performed in the binding sites. 5637 transfection was cotransfected with a mixture of 50 ng FL reporter, 5 ng Renilla luciferase reporter, and 5 pmol miRNA mimic. After48 h, add 100 *μ*L of cell suspension to a 96-well plate, shake slowly on a shaker at room temperature for 15 minutes, aspirate the cell lysate into a 1.5 mL centrifuge tube, centrifuge at 4°C at 12,000 r/min for 10 minutes, take the supernatant, and transfer it into a new tube. Then, the luciferase activity was measured with a dual-luciferase reporter assay system (Promega). The luciferase values were normalized to the corresponding Renilla luciferase values, and then, the fold changes were calculated.

### 2.9. RNA-Binding Protein Immunoprecipitation (RIP)

The RIP lysate was obtained and centrifuged at 12,000 rpm for 10 minutes. RIP assay (Millipore, Billerica, MA) was carried out according to the manufacturer's instructions. Cells were fixed by 1% formaldehyde, lysed, sonicated, and incubated overnight; on the second day, the samples were incubated with 200 ml lysis buffer and protease K to reverse formaldehyde crosslinking. The RNA was purified and obtained from TRIzol. Finally, the immunoprecipitated RNA was detected by RT-PCR.

### 2.10. Fluorescence In Situ Hybridization (FISH)

The tissue is placed in formaldehyde solution, fixed for 24 h, dewaxed, dehydrated, and edge-sealed; the specimen was put into the hybridizer, denatured for 5 min (73°C), and then maintained at a constant temperature of 37°C overnight; the next day, the slices are washed and dried. Observe the slides with a fluorescence microscope in a dark room.

### 2.11. Flow Cytometry Assay for the Cell Cycle

According to the product manual, the transfected plasmid-treated cells were collected and stained with propidium iodide buffer (BD, USA) for cell cycle analysis. The results were analyzed by the ModFit LT software.

### 2.12. Tumor Xenografts

The 4-5-week-old BALB/c nude mice (weight 20–22 g) were randomly divided into 4 groups (shRNA or overexpression hsa_circ_0139402 or negative control or vector control); each group contained 6 mice (total of 24 mice); 2 × 10^7^ cells 5637 were injected into the upper back of BALB/c nude mice and housed in a specific pathogen-free “barrier” facility under controlled temperature (~25°C) and humidity (~50%), with 12 h light/dark cycles. These mice received specific pathogen free (SPF) mouse chow and access to sterile water ad libitum. All animal health and behavior were monitored every 7 days. During the experiment, all animals were healthy, comfortable, nutritious, safe, able to express their nature freely, and free from pain, fear, and stress. One month later, the tumor weight did not exceed 10% of the animal weight. All mice were killed through cervical spondylolysis after injecting 2% sodium pentobarbital anesthetic at the specification of 25 mg/kg. Whether there was respiration and heartbeat after anesthesia was used as the standard of animal death. The tumor volume was recorded after 4 weeks. The volume of tumors was calculated by using the following formula: *V* = *L* × *W*^2^ ÷ 2. The laboratory animals were approved by the medical laboratory animal ethics committee of Jiamusi University. Instructive notions with respect to caring for laboratory animals (which is released by the Ministry of Science and Technology of the People's Republic of China in September 30th, 2006) were followed for the welfare of the animals.

### 2.13. Western Blot Assay

After the cells were washed with PBS, RIPA lysate containing various protease inhibitors was added, the cells were scraped off, the lysate on ice was centrifuged at 4°C, 12,000 rpm for 30 min, and the supernatant was collected. After electrophoresis, transfer the membrane, block, and incubate primary antibody and secondary antibody. After washing with TBST 10 mins for 3 times, the protein was carried out with chemiluminescence development kit (ZATA, USA).

### 2.14. Statistic Analysis

All data were analyzed by SPSS 20.0. *P* < 0.05 indicated statistical significant findings. Data were represented as means ± standard deviation, and Student's *t*-test for two groups and one-way ANOVA with post hoc Bonferroni test for three or more groups were used to assess the statistical significance; the high-throughput sequencing and subsequent bioinformatics analysis were assisted by a gene testing company (biomarker, http://www.biomarker.com.cn/). The Pearson correlation between hsa-miR-326 with hsa_circ_0139402 and PAX8 was calculated to evaluate the reverse expression pattern.

## 3. Results

### 3.1. hsa_circ_0139402 Is Upregulated in BC Patients and Cell Lines

**Figure 1:**
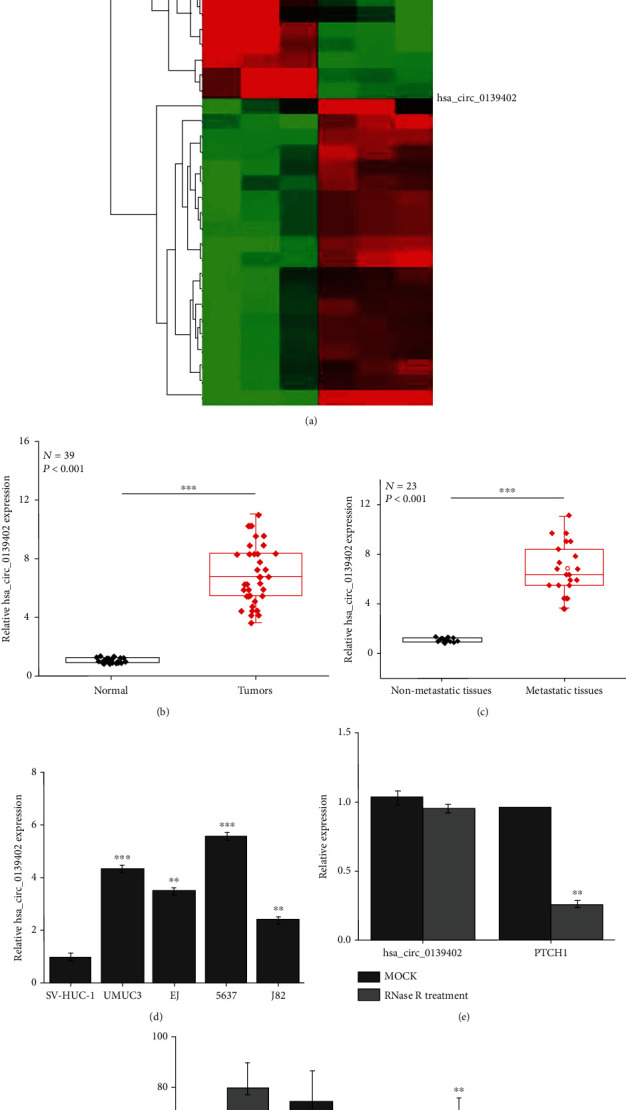
hsa_circ_0139402 is overexpressed in BC patients and cell lines. (a) High-throughput sequencing analysis of the expression of circRNA in 3 pairs of BC tissues; (b) QRT-PCR analysis of the expression levels of hsa_circ_0139402 in BC tissues compared with normal tissues; (c) the expression levels of hsa_circ_0139402 in BC tissues with lymph node metastasis compared with those without metastasis; (d) QRT-PCR analysis of the expression levels of hsa_circ_0139402 in BC cells (UMUC3, EJ, 5637, and J82) and human immortalized uroepithelium cells (SV-HUC-1); (e) QRT-PCR analysis of the expression of hsa_circ_0139402 and mRNA PTCH1 after RNase R treatment in 5637 cells; (f) hsa_circ_0139402 was predominately located in the cytoplasm by FISH. Data were represented as mean ± SD. ^∗^*P* < 0.05 and ^∗∗∗^*P* < 0.001 compared with negative control.

High-throughput sequencing results showed that 295 circRNA has abnormal expression in BC tissues, and the expression of hsa_circ_0139402 was highest in BC tissues (Figure 1(a)); then, RT-PCR result confirmed that the expression of hsa_circ_0139402 was upregulated in 39 pairs of BC tissues and increased in metastatic tissues compared to that of nonmetastatic tissues (32 pairs of BC tissues) (Figures 1(b) and 1(c) and Table 1, ^∗^*P* < 0.05); hsa_circ_0139402 expression was also increased in UMUC3 and 5637. Besides, the expression of hsa_circ_0139402 was significantly increased in UMUC3, UMUC3, 5637, and J82 compared with SV-HUC-1 (Figure 1(d)). Then, after the RNase R treatment, the expression of PTCH1 was significantly decreased; however, the expression of hsa_circ_0139402 had no significant affect (Figure 1(e)), and hsa_circ_0139402 was predominately located in the cytoplasm by FISH (Figure 1(f)).

### 3.2. hsa_circ_0139402 Regulates BC Cells' Proliferation, Migration, and Invasion

**Figure 2:**
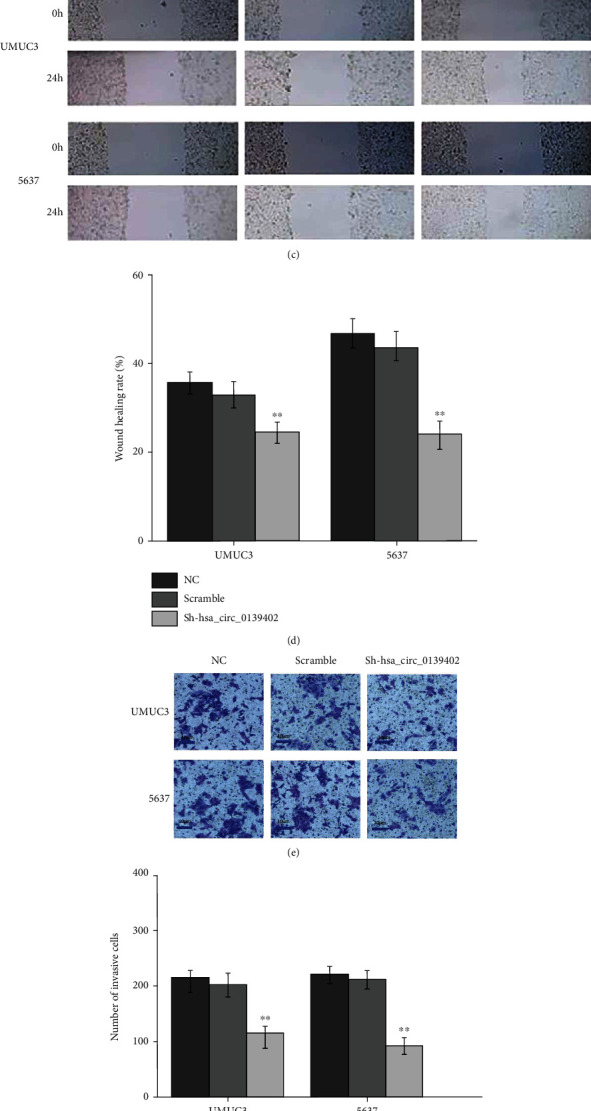
hsa_circ_0139402 regulates BC cells' proliferation, migration, and invasion. (a) sh-hsa_circ_0139402 could regulate the expression of hsa_circ_0139402 in UMUC3 and 5637 cells; (b) MMT assay showed that sh-hsa_circ_0139402 could regulate cell proliferation in UMUC3 and 5637 cells; (c–f) wound healing and transwell assays showed that hsa_circ_0139402 knockdown could regulate migration and invasion in UMUC3 and 5637 cells; (g) hsa_circ_0139402 knockdown affected cell cycle in 5637 cells; (h) BALB/c nude mice injected with 5637 cells cotransfected with sh-hsa_circ_0139402 had smaller volume. Data were represented as mean ± SD. ^∗^*P* < 0.05 compared with negative control.

Then, we want to know if hsa_circ_0139402 affects the function of bladder cancer cell. So sh-hsa_circ_0139402 was transfected into UMUC3 and 5637 cells. The result revealed that sh-hsa_circ_0139402 could significantly decrease the expression of hsa_circ_0139402 in UMUC3 and 5637 cells (Figure 2(a)). However, PTCH1 mRNA expression had no significant change. MTT assay, wound healing assay, and transwell assay revealed that sh-hsa_circ_0139402 could suppress BC cells' proliferation, invasion, and migration (Figures 2(b)–2(f)), and hsa_circ_0139402 knockdown significantly suppressed cell cycle in 5637 cells (Figure 2(g)). BALB/c nude mouse results showed that mice injected with 5637 cells cotransfected with sh-hsa_circ_0139402 had smaller average volume than the control groups (Figure 2(h)).

### 3.3. hsa_circ_0139402 Can Bind to hsa-miR-326, and PAX8 Is a Direct Target of hsa-miR-326 in BC Cell

**Figure 3:**
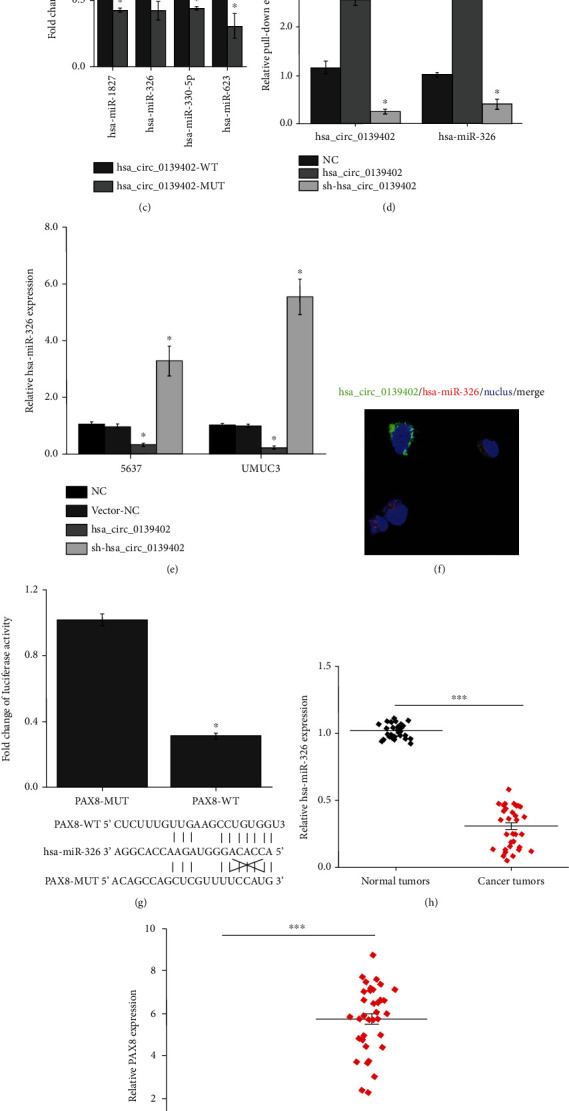
hsa_circ_0139402 acts as a sponge for hsa-miR-326, and PAX8 is a direct target of hsa-miR-326 in BC cell. (a) Bioinformatics analysis was used to search if hsa-miR-326 interacts with hsa_circ_0139402-MUT or hsa_circ_0139402-WT; (b) 4 miRNA mimics were cotransfected with the hsa_circ_0139402 vector into 5647 cells; the line means reduced at least half of the luciferase reporter activities; (c) RNA pull-down assay for the luciferase activity of hsa_circ_0139402-MUT or hsa_circ_0139402-WT in 5637 cells cotransfected with 6 miRNA mimics; (d) RIP assay for the amount of hsa_circ_0139402 and hsa-miR-326 in 5637 cells transfected with hsa_circ_0139402 overexpression or sh-hsa_circ_0139402 or negative control; (e) qRT-PCR analysis of expression levels of hsa-miR-326 in 5637 and UNUC3 cells transfected with hsa_circ_0139402 overexpression or sh-hsa_circ_01394021 or negative control; (f) immunofluorescence stain shows the interaction of hsa-mir-326 with hsa_circ_0139402; (g) luciferase reporter assay for the luciferase activity of PAX8-MUT or PAX8-WT in 5637 cells cotransfected with hsa-miR-326; (h) QRT-PCR analysis of the expression levels of hsa-miR-326 in BC tissues compared with normal tissues; (i) the expression levels of PAX8 in BC tumor tissues compared with adjacent normal tissues; (j) Pearson correlation was used for correlation analysis between hsa_circ_0139402, hsa-miR-326, and PAX8 in BC patients. Data were represented as mean ± SD. ^∗^*P* < 0.05 compared with negative control; ^#^*P* < 0.05 compared with hsa_circ_0139402+hsa-miR-326 mimics.

To investigate the underlying mechanisms of hsa_circ_0139402 that suppressed cell proliferation, invasion, and migration, four publicly online tools, circbase (http://circrna.org/), circinteractome (https://circinteractome. http://nia.nih.gov/), starbase V3.0 (http://starbase.sysu.edu.cn/), and circbank (http://www.circbank.cn/index.html), were used to predict the possible binding miRNAs of hsa_circ_0139402, and 4 potential miRNAs were picked (Figures 3(a) and 3(b)). Dual-luciferase reporter assay revealed that 4 miRNAs (hsa-miR-1827, hsa-miR-326, hsa-miR-330-5p, and hsa-miR-623) could decrease luciferase reporter activities (Figure 3(c)). In addition, we used biotin-labeled hsa_circ_0139402 probe which was copurified with hsa-miR-326 more than the NC group (Figure 3(d)). The expression levels of hsa-miR-326 were upregulated when transfected with sh-hsa_circ_0139402 plasmid and decreased by hsa_circ_0139402 overexpression plasmid in UMUC3 and 5637 cells (Figure 3(e)). FISH assay showed that in the cytoplasm, hsa_circ_0139402 was colocalized with hsa-miR-326 (Figure 3(f)).

The starbase V3.0 database (http://starbase.sysu.edu.cn/) was used to predict the target genes of hsa-miR-326. Dual-luciferase reporter assay confirmed that the luciferase activity was decreased by the PAX8-WT group but not the PAX8-MUT group (Figure 3(g)). RT-PCR revealed that the expression of hsa-miR-326 was reduced, and PAX8 was significantly increased in BC tumor tissues compared with normal tissues (Figures 3(h) and 3(i)). In addition, correlation analysis showed a moderately negative correlation between hsa-miR-326 with hsa_circ_0139402 and PAX8 and a moderately positive correlation between hsa_circ_0139402 and PAX8 (Figure 3(j)).

### 3.4. hsa_circ_0139402 Promotes Bladder Cells' Proliferation, Migration, Invasion, and EMT by Targeting PAX8 via hsa-miR-326

**Figure 4:**
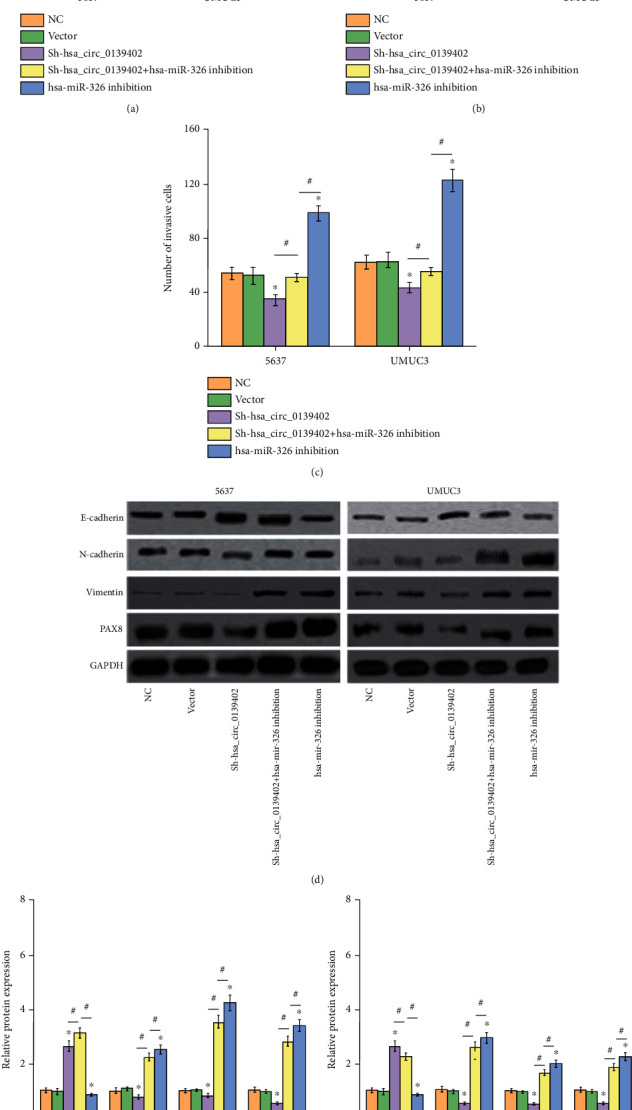
hsa_circ_0139402 inhibits bladder cells' proliferation, migration, invasion, and EMT by targeting PAX8 via hsa-miR-326. (a) CCK8 assay showed that negative control or sh-hsa_circ_0139402 or sh-hsa_circ_0139402+hsa-miR-326 inhibition or hsa-miR-326 inhibition could regulate 5637 and UMUC3 cells' proliferation; (b) wound healing assay showed that negative control or sh-hsa_circ_0139402 or sh-hsa_circ_0139402+hsa-miR-326 inhibition or hsa-miR-326 inhibition could regulate 5637 and UMUC3 cells' migration; (c) transwell assay showed that negative control or sh-hsa_circ_0139402 or sh-hsa_circ_0139402+hsa-miR-326 inhibition or hsa-miR-326 inhibition could regulate 5637 and UMUC3 cells' invasion; (d–f) western blot analysis showed that the expression levels of PAX8, E-cadherin, N-cadherin, and Vimentin transfected with sh-hsa_circ_0139402+hsa-miR-326 inhibition or hsa-miR-326 inhibition could regulate 5637 and UMUC3 cells. Data were represented as mean ± SD. ^∗^*P* < 0.05; ^#^*P* < 0.05 compared with sh-circPVT1+miR-140-3p inhibition.

This study also investigated whether or not hsa_circ_0139402 promotes bladder cancer cell proliferation, migration, and invasion by regulating hsa-miR-326/PAX8 signaling. Those results showed that the hsa-miR-326 inhibition group could reverse the function of BC cells' proliferation, migration, and invasive cotransfection with sh-hsa_circ_0139402 (Figures 4(a)–4(c)). WB showed that the expression of PAX8 protein had similar results in BC cells. Furthermore, E-cadherin expression was significantly decreased, N-cadherin and Vimentin were increased by sh-hsa_circ_0139402, and hsa-miR-326 inhibition could significantly decrease E-cadherin expression and increase N-cadherin and Vimentin expression (Figure 4(d)); however, hsa-miR-326 inhibition could neutralize the expression of EMT biomarker cotransfected with sh-hsa_circ_0139402 in BC cells (Figures 4(e) and 4(f)).

## 4. Discussion

Bladder cancer is a disease with high morbidity, high mortality, and high treatment costs [14, 15]. In the past 10 years, with the continuous in-depth research on the molecular mechanism of tumorigenesis, a variety of biomarkers related to tumorigenesis have been proved to be molecular markers for diagnosis and prognosis. Among them, circRNA is a hot topic in recent years, and its abnormal expression exists in many kinds of tumors including bladder cancer, such as lung adenocarcinoma, colorectal cancer, and hepatocellular carcinoma [16–20]. Our study showed that high-throughput sequencing results showed that the expression of hsa_circ_0139402 was highest in BC tissues,

Studies have confirmed that miR-326 exerts a tumor suppressor effect by inhibiting cell proliferation, migration, and invasion and inducing cell apoptosis [21–28]. As a tumor suppressor, mir-326 is involved in the formation and progress of non-small-cell lung cancer, esophageal squamous cell carcinoma, and glioblastoma [29–32]. For example, miR-326 can significantly inhibit the proliferation and invasion of NSCLC cells, esophageal squamous cancer cells, and glioblastoma. It can be seen that miR-326 is expected to become a potential biomarker and therapeutic target for a variety of tumors.

Studies have reported that EMT is the driving force for cancer cells' metastasis [33–35]. EMT is an important mechanism in the early stage of cancer metastasis, and to study the molecular mechanism of EMT is the key to improve tumor diagnosis and treatment. The decreased expression of E-cadherin and Vimentin, N-cadherin, and other proteins has increased which has been considered to be the most significant feature of EMT. The changes of these EMT markers have enabled cancer cells to obtain features that promote migration and invasion. Similarly, our results reveal that hsa_circ_0139402 could regulate the E-cadherin/N-cadherin pathway to inhibit the EMT in BC cells.

## 5. Conclusion

In conclusion, this study revealed that the expression of hsa_circ_0139402 is more highly in BC tissues and cell lines, and hsa_circ_0139402 promotes bladder cancer progression by regulating hsa-miR-326/PAX8 signaling. Our results suggest that hsa_circ_0139402 may be a novel potential biomarker in BC.

## Figures and Tables

**Table 1:** Correlation between clinicopathological characteristics and expression of hsa_circ_0139402 in 39 bladder cancer and matched normal adjacent tissue.

Characteristic	Cases (*n* = 39)	Relative expression		*P* value
		High	Low	
Gender				
Male	27	18	9	1.000
Female	12	8	4	
Ages				
≤65	16	7	9	0.209
>65	23	15	8	
Lymphatic metastasis				
Positive	23	19	4	0.017^∗^
Negative	16	7	9	
Tumor size (cm)				
<3	11	5	6	0.307
>3	28	18	10	
TNM stage				
I-II	20	14	6	0.741
III-IV	19	12	7	
Grade				
Low	18	13	5	0.812
High	21	15	6	

## Data Availability

All data generated or analyzed during this study are included. Further inquiries can be directed to the corresponding author.

## References

[1] Sung H., Ferlay J., Siegel R. L. (2021). Global cancer statistics 2020: GLOBOCAN estimates of incidence and mortality worldwide for 36 cancers in 185 countries. *CA: a Cancer Journal for Clinicians*.

[2] Meng S., Zhou H., Feng Z. (2017). CircRNA: functions and properties of a novel potential biomarker for cancer. *Molecular Cancer*.

[3] Zhang Z., Yang T., Xiao J. (2018). Circular RNAs: Promising Biomarkers for Human Diseases. *eBioMedicine*.

[4] Chen L. L. (2020). The Nature Reviews. *Molecular Cell Biology*.

[5] Li X., Yang L., Chen L. L. (2018). The Biogenesis, Functions, and Challenges of Circular RNAs. *Molecular Cell*.

[6] Wei Y., Zhang Y., Meng Q., Cui L., Xu C. (2019). Hypoxia-induced circular RNA has_circRNA_403658 promotes bladder cancer cell growth through activation of LDHA. *American Journal of Translational Research*.

[7] Zhang L., Hou C., Chen C. (2020). The role of N6-methyladenosine (m6A) modification in the regulation of circRNAs. *Molecular Cancer*.

[8] Chen C., Huang Z., Mo X. (2020). The circular RNA 001971/miR-29c-3p axis modulates colorectal cancer growth, metastasis, and angiogenesis through VEGFA. *Journal of Experimental & Clinical Cancer Research*.

[9] Su Y., Feng W., Shi J., Chen L., Huang J., Lin T. (2020). circRIP2 accelerates bladder cancer progression via miR-1305/Tgf-*β*2/smad3 pathway. *Molecular Cancer*.

[10] Dong W., Bi J., Liu H. (2019). Circular RNA ACVR2A suppresses bladder cancer cells proliferation and metastasis through miR-626/EYA4 axis. *Molecular Cancer*.

[11] Liu F., Zhang H., Xie F. (2020). Hsa_circ_0001361 promotes bladder cancer invasion and metastasis through miR-491-5p/MMP9 axis. *Oncogene*.

[12] Han J., Wang J. Z., Yang X. (2019). METTL3 promote tumor proliferation of bladder cancer by accelerating pri-miR221/222 maturation in m6A-dependent manner. *Molecular Cancer*.

[13] Chi B. J., Sun Y., Zhao J. T., Bi S., Wang S. Q., Huang L. (2020). CircPVT1 promotes bladder cancer progression by acting as a ceRNA for miR-140-3p to target TRPS1. *Researchsquare*.

[14] Cheng Y., Nie S., Li L. (2019). Epidemiology and outcomes of acute kidney injury in hospitalized cancer patients in China. *International Journal of Cancer*.

[15] Amato L., Fusco D., Acampora A. (2017). Volume and health outcomes: evidence from systematic reviews and from evaluation of Italian hospital data. *Epidemiologia e prevenzione*.

[16] Zeng K., Chen X., Xu M. (2018). CircHIPK3 promotes colorectal cancer growth and metastasis by sponging miR-7. *Cell Death & Disease*.

[17] Chen Q., Liu T., Bao Y. (2020). CircRNA cRAPGEF5 inhibits the growth and metastasis of renal cell carcinoma via the miR-27a-3p/TXNIP pathway. *Cancer Letters*.

[18] Wang C., Tan S., Li J., Liu W. R., Peng Y., Li W. (2020). CircRNAs in lung cancer - Biogenesis, function and clinical implication. *Cancer Letters*.

[19] Kristensen L. S., Hansen T. B., Venø M. T., Kjems J. (2018). Circular RNAs in cancer: opportunities and challenges in the field. *Oncogene*.

[20] Li Y., Zhao J., Yu S. (2019). Extracellular vesicles long RNA sequencing reveals abundant mRNA, circRNA, and lncRNA in human blood as potential biomarkers for cancer Diagnosis. *Clinical Chemistry*.

[21] Ji S., Zhang B., Kong Y., Ma F., Hua Y. (2017). miR-326 inhibits gastric cancer cell growth through downregulating NOB1. *Oncology Research*.

[22] Zhang J., He H., Wang K. (2020). miR-326 inhibits the cell proliferation and cancer stem cell-like property of cervical cancer in vitro and oncogenesis in vivo via targeting TCF4. *Annals of Translational Medicine*.

[23] Nie F. R., Li Q. X., Wei H. F., Ma Y. (2020). miR-326 inhibits the progression of papillary thyroid carcinoma by targeting MAPK1 and ERBB4. *Neoplasma*.

[24] Liu W., Zhang B., Xu N., Wang M. J., Liu Q. (2017). miR-326 regulates EMT and metastasis of endometrial cancer through targeting TWIST1. *European Review for Medical and Pharmacological Sciences*.

[25] Cai L., Chen J. J., Deng F. M., Wang L., Chen Y. (2021). MiR-326 regulates the proliferation and apoptosis of endometrial cancer by targeting Bcl‐2. *Journal of Obstetrics and Gynaecology Research*.

[26] Zhang J., Wei X., Zhang W., Wang F., Li Q. (2020). MiR-326 targets MDK to regulate the progression of cardiac hypertrophy through blocking JAK/STAT and MAPK signaling pathways. *European Journal of Pharmacology*.

[27] Wang R., Xu J., Xu J. (2019). MiR-326/Sp1/KLF3: a novel regulatory axis in lung cancer progression. *Cell Proliferation*.

[28] Pan Y. J., Wan J., Wang C. B. (2019). MiR-326: promising biomarker for Cancer. *Cancer Management and Research*.

[29] Wei J., Meng G., Wu J. (2021). MicroRNA-326 impairs chemotherapy resistance in non small cell lung cancer by suppressing histone deacetylase SIRT1-mediated HIF1*α* and elevating VEGFA. *Bioengineered*.

[30] Li D., Du X. U. S. H. E. N. G., Liu A., Li P. (2016). Suppression of nucleosome-binding protein 1 by miR-326 impedes cell proliferation and invasion in non-small cell lung cancer cells. *Oncology Reports*.

[31] Huang L., Wang Y., Chen J. (2019). Long noncoding RNA PCAT1, a novel serum-based biomarker, enhances cell growth by sponging miR-326 in oesophageal squamous cell carcinoma. *Cell Death & Disease*.

[32] Yin Y., Tan Y., Yao Y., Lu N., Zhang F. (2020). SNHG12/miR-326/E2F1 feedback loop facilitates the progression of oral squamous cell carcinoma. *Oral Diseases*.

[33] Hong D., Fritz A. J., Zaidi S. K. (2018). Epithelial-to-mesenchymal transition and cancer stem cells contribute to breast cancer heterogeneity. *Journal of Cellular Physiology*.

[34] Jiang J., Wang K., Chen Y., Chen H., Nice E. C., Huang C. (2017). Redox regulation in tumor cell epithelial-mesenchymal transition: molecular basis and therapeutic strategy. *Signal Transduction and Targeted Therapy*.

[35] Kumar S., Mehta K. (2013). Tissue transglutaminase, inflammation, and cancer: how intimate is the relationship?. *Amino Acids*.

